# Microbiota–host interactions shape ageing dynamics

**DOI:** 10.1098/rstb.2019.0596

**Published:** 2020-08-10

**Authors:** Miriam Popkes, Dario Riccardo Valenzano

**Affiliations:** 1 Max Planck Institute for Biology of Ageing, Cologne, Germany; 2 CECAD, University of Cologne, Cologne, Germany

**Keywords:** microbiota, ageing, longevity, immune system, adaptive immunity

## Abstract

Occupying the interface between host and environment, host-associated microbes play fundamental roles in nutrient absorption, essential metabolite synthesis, development of the immune system, defence against pathogens and pathogenesis. Microbiota composition and function is rather stable during adulthood, while it dramatically changes during early development, frailty and disease. Ageing is associated with progressive decrease of homeostasis, often resulting in disruption of the physiological balance between host and commensal microbes, ultimately leading to dysbiosis and host demise. Generally, high microbial diversity is associated with health and a youthful state, while low individual microbial diversity and larger inter-individual microbial diversity is associated with ageing and disease states. Different species are equipped with species-specific commensal, symbiotic and pathogenic microbial communities. How and whether the specific host–microbiota consortia co-evolved with host physiology to ensure homeostasis and promote individual fitness remains an open question. In this essay, we propose that the evolution of vertebrate-specific immune adaptations may have enabled the establishment of highly diverse, species-specific commensal microbial communities. We discuss how the maintenance of intact immune surveillance mechanisms, which allow discrimination between commensal and pathogenic bacteria, fail during ageing and lead to the onset of known ageing-related diseases. We discuss how host–microbiota interactions are key to maintaining homeostasis despite external perturbations, but also how they affect a range of host-specific ageing-related phenotypes.

This article is part of the theme issue ‘The role of the microbiome in host evolution’.

## 1. Introduction

The evolution of the adaptive immune system provided vertebrates with a novel molecular and cellular toolbox to deploy efficient and long-lasting defence responses against parasites and pathogens. At the same time, the emergence of lymphocyte-based adaptive immunity was instrumental to the establishment of host-specific, highly complex commensal microbial communities. Whether also microbes that are part of commensal microbiota (e.g. immune-modulating microbes in the mammalian intestines) co-evolved with the host is still an open question. Highly diverse microbial communities associated with vertebrate epithelia gave access to a staggeringly vast range of microbial metabolites and molecular intermediates, allowing for a significant upgrade of the biosynthetic pathways encoded by the host's genome alone. However, while the establishment of a complex microbiota composition led to the acquisition of a large set of significant advantages, it also came with new costs. Imbalance in the complex signalling pathways that regulate the adaptive immune system can lead to dysfunctional responses towards pathogens and commensals, ultimately resulting in disease and death. The significant changes in microbiota composition occurring during ageing could thus be a direct consequence of the long evolutionary history shared between vertebrates and their complex, species-specific microbial communities.

## 2. Host–microbiota interactions have a long evolutionary history

**Figure 1.**
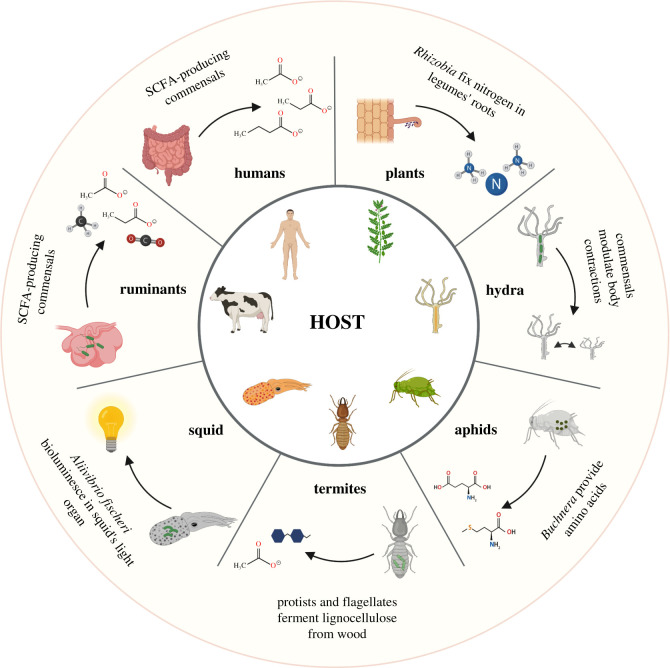
Host-specific microbiota. Coevolution of microbes and multicellular hosts leads to mutualistic relationships. The host builds a dynamic ecological niche which provides nutrients and a stable environment to the microbes. The microbiota, in turn, provide nutrients and novel metabolic pathways. Clockwise, from top right: legume roots establish symbiotic interactions with *Rhizobia* bacteria in the soil, which fix nitrogen to molecular forms accessible to the plant. Species-specific microbiota in the hydra modulate spontaneous body contractions and prevent lethal fungal infections. In sap-feeding aphids, endosymbiotic *Buchnera* provide the host with essential amino acids lacking in the sap. Protists and flagellates in termites ferment lignocellulose from wood. The bobtail squid hosts symbiotic colonies of bioluminescent *Aliivibrio fischeri* in its light organ, helping with defence and hunting behaviours. In ruminants, cellulose-fermenting bacteria digest fibre-rich plants into host-accessible metabolites, such as short-chain fatty acids (SCFA). Commensal microbes in the human intestine provide nutrients like SCFA, secondary bile acids and essential vitamins. This figure was generated with Biorender.

Microbial communities have inhabited our planet for over 3 billion years, long before the emergence of multicellular life. Endosymbiosis was key for the origin of eukaryotes, leading to the evolution of organelles, enabling cellular structural and functional compartmentalization [1–3], and providing host cells with new metabolic pathways. Given the ubiquity of microbes in the environment, eukaryote evolution happened within the context of a microbial biosphere. The interdependence between multicellular hosts and specialized microbiota, however, goes far beyond the intracellular level, spanning the whole tree of life (figure 1). Simple aquatic multicellular organisms, such as sponges and Cnidaria, are immersed in a vast and diverse microbial ecosystem, and their biology is strictly dependent on these communities. Mutualistic interactions between bacteria and simple or complex eukaryotes have repeatedly evolved over time, providing benefits to both sides. The profound ecological and physiological interdependence between multicellular organisms and prokaryotes have led to the emergence of the concept of the metaorganism or holobiont [4]. The microbial–host mutual interactions are not unique to simple aquatic organisms. Terrestrial invertebrates, such as sap-feeding aphids, need endosymbiont Proteobacteria of the genus *Buchnera* to synthesize essential amino acids, which are lacking in the sap that aphids feed on. In return, aphids provide a stable ecological niche to their bacterial partner. This partnership is obligate for either side, as it is necessary for reproduction and survival [5]. Obligate symbioses occur frequently among insects, like in the charismatic case of leafcutter ants of the genera *Atta* and *Acromyrmex,* which strictly depend on their domesticated fungal cultivars as food source [6]. Paradigmatic cases of host–microbe coevolution are those of bobtail squids, hosting bioluminescent *Aliivibrio fischeri*, which allow squids to perform complex nocturnal defence and hunting behaviours [7]; and termites, which host specialized gut-dwelling protists and flagellates that play essential roles in food digestion [8]. Similar to termites, ruminants strictly depend on their cellulose-fermenting microbes to digest fibre-rich plants, which are hosted in specialized fermenting chambers, i.e. highly adapted anatomical structures, such as the rumen [9].

**Figure 2.**
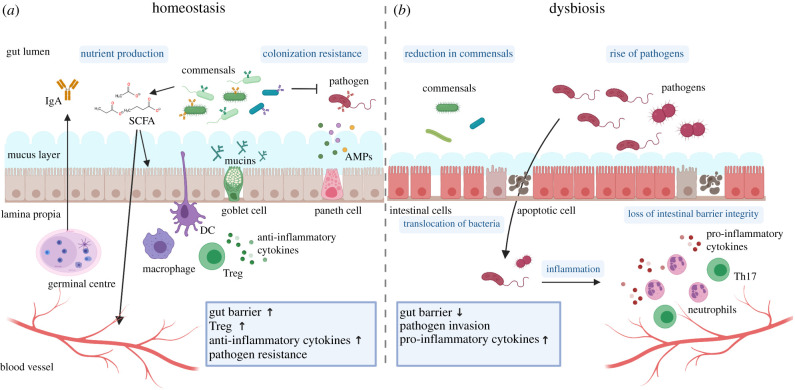
Host–microbiota interactions under homeostatic and dysbiotic conditions. (*a*) Under homeostatic conditions, the intestine shows a well-balanced interplay between host and microbiota. The host allows specific microbes to reside in the lumen, which in turn provide nutrients, such as short-chain fatty acids (SCFA), and help the defence against pathogens contributing to colonization resistance. To ensure proper homeostasis, the host actively selects for specific commensal bacteria—resulting in a diverse commensal community—while keeping the bacteria at a safe distance through the intestinal gut barrier. The gut barrier is composed of a thin layer of epithelial cells, a thick mucus barrier and defence molecules, such as anti-microbial peptides (AMPs). Mucin production by goblet cells and barrier function is enhanced by the bacterial-derived SCFA. Plasma cells in the lamina propria or the germinal centres (GC) produce secretory IgA, which coat both commensal and pathogenic bacteria. Intestinal macrophages produce large amounts of anti-inflammatory cytokines that block pro-inflammatory signals and promote regulatory T cells (Treg), which help maintain immune homeostasis in the intestine. (*b*) Under dysbiosis and ageing, the intestinal microbiota composition undergoes a reduction of commensal and a rise of pathogenic bacteria. Loss of intestinal barrier integrity enables translocation of bacteria into the host tissue, through the basement membrane into the lamina propria. Pathogen invasion results in a recruitment of inflammation-associated immune cells like neutrophils and Th17 cells, leading to a burst of pro-inflammatory cytokines. This figure was generated with Biorender.

The ubiquitous occurrence of multicellular host–microbiota mutualism is made possible in extreme cases by the presence of highly sophisticated anatomical compartments, such as specialized light organs in bobtail squids, light organs in flashlight fish and rumens in ruminants. However, to establish commensal interactions with microbial partners, the vast majority of organisms rely on specialized epithelia, integuments and mucosal membranes, which ensure physical separation between the inside and the outside. These specialized membranes and their highly heterogeneous cellular composition are capable of protection, but also ensure the key functions of recognition and molecular cross-feeding between microbes and their multicellular hosts [10,11]. The sophistication of the epithelia–microbiota molecular cross-talk reaches peaks of complexity in the large intestines of vertebrates, where mucosal immunity protects the host from pathogens and fosters a diverse community of commensals (figure 2).

Highly complex microbial communities, being similar among members of the same species, define species-specific microbiota. For instance, the lineage leading to humans, gorilla, chimpanzee and bonobos presents species-specific microbial assemblies, where specific anatomical, physiological and behavioural adaptations in the host are associated with specialized microbial commensal communities, possibly reflecting a long coevolutionary history between hosts and microbes [12].

But how do hosts become associated with specific microbial communities? The establishment of species-specific, complex microbiota communities during life—for instance in vertebrate intestines—is a process of dynamic niche construction [13], in which the host actively shapes its own environment by promoting beneficial microbes and suppressing opportunistic pathogens. Commensal microbes, in turn, contribute to host homeostasis by promoting nutrient absorption and protecting the host from pathogens [14]. Additionally, commensals cross-feed metabolites with other microbes, further contributing to build their own microbial niche [15]. A typical example of niche construction is the establishment of the infant microbiota community in humans through maternal breastmilk feeding. After lipids and lactose, breastmilk is largely composed of human milk oligosaccharides (HMOs), which are not absorbed by newborns [16]. HMOs in the offspring gut represent a specific food source to beneficial *Bifidobacteria* [17]. While direct placenta-mediated vertical transmission of the microbiota in humans has been questioned [18], mothers contribute to shaping their offspring's microbial niche construction through a range of mechanisms, including maternal antibodies transmitted to the offspring (IgA and IgG), which directly modulate CD4^+^ T cells, dampening responses to neonatal bacteria [19] and helping the newly established microbiota community to take shape. Commensal microbes provide hosts with immune modulatory molecular signals, such as short-chain fatty acids (SCFA, a product of the metabolism of dietary fibres by anaerobic bacteria), which can help promoting anti-inflammatory responses [20]. Moreover, they cross-feed other bacteria, leading to the establishment of a diverse commensal microbial community [21]. Complex commensal microbial communities associated with healthy hosts have been shown to help the host defeat pathogens, e.g. by resisting colonization and competing for resources [22,23]. Given their active role in the maintenance of homeostasis in their host, commensal microbes can be considered as key players in host immunity.

The assembly of individual (e.g. gut-specific) microbiota also depends on external factors, such as temperature, nutrients, microbes available through food and via contact (e.g. directly transmitted) with conspecifics [24,25]. Other factors important in shaping microbiota communities are the stomach pH, the oxygen concentration, the length and cellular composition of different portions of the gastrointestinal tract, and the molecular composition of the mucus layer that lines vertebrate's intestines [26,27]. Microbiota associated with host-specific diets, e.g. a carnivore or herbivore lifestyle, which contain genes with diet-specific enzymatic capacities, are moulded by the presence of specific anatomical adaptations of the host's gastrointestinal tract (a longer or shorter intestine, the presence of the rumen, etc.) [28–30]. In humans, the early acquisition of a commensal microbial community is strictly dependent on external factors, such as modality of delivery (caesarean versus vaginal) [31], feeding on maternal breast milk or formula, age at which the ingestion of solid food starts, geographical location, the presence of siblings and others [32]. During adult life, diet provides both the microbes and the nutrients that help shape the composition of the microbiota [33,34]. Different human populations, eating each their own food, largely differ in their gut microbial species richness and composition [35], and populations with similar lifestyles—e.g. either traditional or industrialized societies—tend to share same members of the microbiota (e.g. same taxonomic families). Noteworthy, in healthy humans, genetics seems to play a lesser role in shaping the composition of the intestinal microbiota, compared to diet [36].

## 3. Adaptive immune system and the establishment of a diverse commensal microbiota

Innate immunity is an ancient line of defence against pathogens, shared among multicellular organisms, including plants and invertebrates [37,38]. The innate immune system prevents infections by increasing barrier function via physical separation, secretion of antimicrobial peptides (AMP) and phagocytosis. Innate immunity evolved to recognize and respond automatically to a set of pre-determined microbial ligands, in response to which it elicits inflammatory and anti-viral responses [39]. Specialized receptors, including Toll-like receptors, NOD-like receptors and C-type lectin receptors, also collectively known as pattern recognition receptors, identify conserved molecular patterns and thus detect pathogens or defective cell components [40–42]. By delivering effective defence responses against common pathogens, e.g. through AMP, innate immunity shapes simple and host-specific commensal microbial communities [43,44].

Early vertebrates evolved a novel form of immunity, which uses an extremely diverse set of receptors to map the antigenic variability of the environment (both internal and external) far beyond the limits reached by the innate immune system [45]. While jawless vertebrates evolved leucine rich repeat based lymphocyte receptors as a system to generate heterogeneous and evolving receptors [46], jawed vertebrates evolved a system based on immunoglobulins and T cell receptors [45]. Adaptive immunity in both jawless and jawed vertebrates employed systems of hypermutation, gene conversion and somatic recombination to generate this novel set of cell-specific receptors [47], permitting a sophisticated defence mechanism against repeated immunological threats, such as viral, microbial and fungal infections. By employing a system of pathogen recognition and immunological memory, lymphocyte-based adaptive immunity leads to targeted and powerful responses against previously encountered pathogens [48]. Alongside the evolution of sophisticated molecular mechanisms that enable recognition and neutralization of fast evolving pathogens (including viruses and diverse bacteria), adaptive immunity allowed a range of mechanisms favouring immune tolerance for diverse communities of microbial commensals [49]. A lymphocyte-based adaptive immunity favoured immune tolerance of commensal microbes by preventing or suppressing automatic innate immune responses against both strain-specific microbial antigens [50], as well as by neutralizing the pro-inflammatory cascades induced by general microbial antigens, such as lipopolysaccharides, which are constitutive components of the external membrane of Gram-negative bacteria [51]. Secreted immunoglobulins A (IgA), the most predominant antibody class in mucosal surfaces, play a key role in maintaining the epithelium–microbiota balance. IgA bind microbial-derived molecules (e.g. toxins) preventing their absorption in the epithelium, coat microbes directly to prevent their growth, and induce a number of downstream molecular cascades in myeloid effector cells, which mediate both pro- and anti-inflammatory responses [52,53]. The occupation of mucosal intestinal surfaces by members of the healthy commensal microbiota has been shown to be dependent on IgA responses [54]. The heterogeneity in the effector functions induced by different classes of IgA depends in part on the glycosylation profiles of the Fc region of the antibody [55]. Together with IgA, IgM antibodies also play an important role in shaping a diverse microbial community in association with the mucus layer of the mammalian intestinal epithelium [56]; and recently IgD have also been proposed as potential players in establishing a symbiotic interaction between host and microbiota [57].

T lymphocytes also help maintaining the host–microbial balance in mucosal organs through the action of specialized subclasses of helper cells (e.g. T_regs_) that respond to commensal antigens by expressing anti-inflammatory cytokines and by dampening pro-inflammatory programmes in effector cells [58]. Furthermore, cytotoxic T cells can favour commensal microbes by selectively eliminating other effector cells [59].

Microbes can protect themselves from immune attacks within the gastrointestinal tract through the synthesis of specific microbial intermediates (e.g. short-chain fatty acids (SCFA)), which dampen pro-inflammatory responses both locally (e.g. in colonocytes and more broadly on the gut epithelium), as well as systemically, altering glucose metabolism and T cell immune responses [60].

Through the evolution of sophisticated molecular signalling between microbes and the host adaptive immune system, hosts provide a stable ecological niche to highly diverse commensal microbial communities. These host-associated microbiota, in turn, provide access to a staggering variety of enzymatic reactions, which result in novel bioactive molecules and metabolites that largely exceed the host's metabolic range [61]. Throughout evolution, microbiota-derived molecules have become essential to host physiology, contributing to fundamental biological functions, including development, growth and maintenance of homeostatic processes. Essential vitamins, such as cobalamin (vitamin B_12_), folic acid (vitamin B_9_), biotin, as well as secondary bile acids, are key products of microbiota metabolism [62]. Additionally, gut microbes are able to synthesize neurotransmitters, such as serotonin, dopamine and GABA [63]; however, whether these neurotransmitters are uniquely active in the enteric nervous system or whether they also act in the central nervous system is not yet fully understood. The sophisticated molecular cross-talk between microbiota and host immune system suggests the possibility of their coevolution. The physiological dependence of host metabolism on microbial communities and the emergence of vertebrate adaptive immune system—itself largely shaped by commensal bacteria [64]—may have been the key innovation to enable complex host–microbiota functional integration.

However, if on the one hand adaptive immunity enabled hosts to control complex commensal microbial communities, with great benefits to the host, on the other hand it also created novel chances for homeostatic failure, for instance when the well-balanced host–microbiota interaction becomes compromised, such as during autoimmune diseases and ageing.

## 4. Ageing and dysbiosis

Ageing is a shared feature among nearly all living organisms, and is characterized by the age-dependent decline of virtually all homeostatic functions. Ageing-dependent dysfunction scales at all levels of biological complexity, from accumulation of DNA and protein damage in cells, to organelle dysfunction (e.g. mitochondrial dysfunction), to cellular senescence, organ disbalance owing to altered cellular composition and irreversible extracellular matrix modifications [65]. Age-related metabolic and cellular changes occur alongside systemic and chronic low-grade inflammation, called inflammaging [66]. Homeostatic disbalance and declined immune system function correlates with chronic inflammation, increase in the rate of infectious diseases, as well as degenerative diseases, such as arteriosclerosis, type 2 diabetes, cancer and Alzheimer's disease, among others [67]. Cumulatively, the ageing-dependent systemic decline leads to the progressive increase in the risk of death [68].

**Figure 3.**
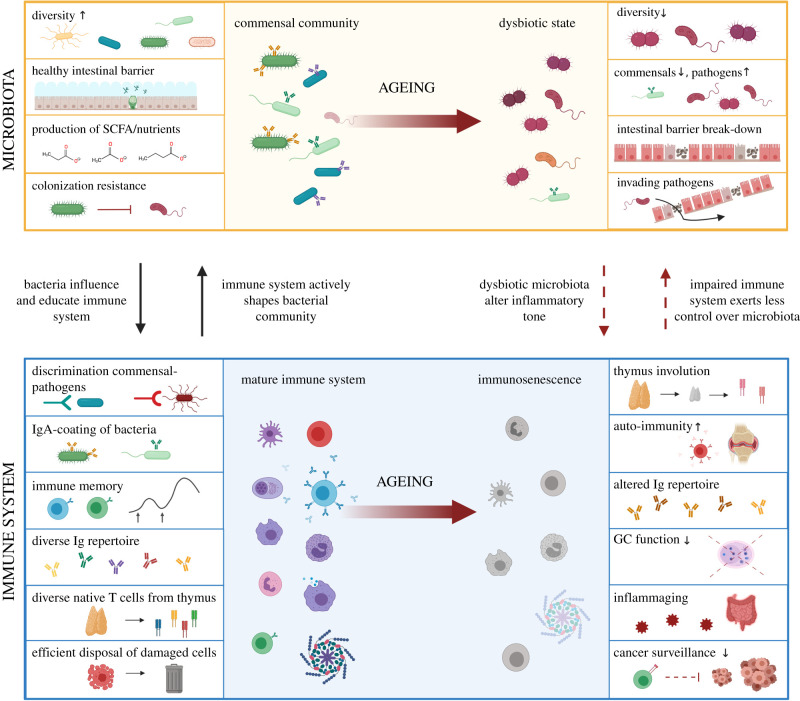
Loss of immune-microbiota balance during ageing. Top left: a young and healthy host is characterized by a diverse commensal microbial community. SCFA produced by microbes enhance the intestinal barrier function and act as potent immune modulators. Commensal microbes prevent growth of pathogens providing colonization resistance. The intestinal microbiota as a whole contributes to the development of the mature immune system. Bottom left: the adaptive immune system helps discriminate between commensal and pathogenic bacteria, actively shaping the microbial community by neutralizing pathogenic microbes and creating immune tolerance towards beneficial bacteria. Innate immune cells, together with T cells, ensure proper disposal of damaged, senescent and cancerous cells. Top right: during ageing, dysbiotic microbial communities affect the inflammatory tone in the intestine and the impaired immune system exerts less control over intestinal microbiota. The bacterial diversity in the intestine decreases, with a decline in commensals and a rise of pathogens. Intestinal barrier breakdown can lead bacteria to invade host tissues, resulting in inflammatory responses. Bottom right: ageing of the immune system (immunosenescence) consists of several processes, such as thymus involution, which leads to a reduced output of naive T cells. T cell receptor (TCR) diversity is consequently decreased and the TCR repertoire is biased towards autoreactive cells, possibly contributing to autoimmunity. The immunoglobulin repertoire diversity is altered, impairing responses to novel immune challenges (e.g. new pathogenic microbes or vaccinations). Germinal centres (GC), playing a major role in IgA production, decrease functionality during ageing. Dysbiosis and intruding pathogens during ageing probably contribute to age-related chronic low-grade inflammation, called ‘inflammaging’. Ageing-related low-grade inflammation promotes cancer progression, exacerbated by declined immune surveillance by aged lymphocytes. This figure was generated with Biorender.

Recent evidences indicate that the extensive molecular changes occurring during host ageing may significantly impact the balance between the host and its epithelia-dwelling commensal microbial communities (figure 3) [69–71]. The disruption of the homeostatic balance between microbiota and the host can result in the so-called ‘dysbiosis’ which characterizes several diseases, including cancer and autoimmune conditions, such as intestinal bowel syndrome [72], Crohn's disease [73] and many others. Intestinal dysbiosis has been connected with immune-deficiencies (e.g. HIV infections) [74] and with several neurodegenerative conditions leading to cognitive impairment, e.g. in neurological and psychiatric diseases [75]. Ageing in vertebrates' intestines co-occurs with the alteration of intestinal microbiota communities, marked by a distinct shift in composition and decrease in within-individual microbial richness [76–78]. During ageing, the relative abundance of microbial taxa typically associated with a young-adult, ‘healthy’ state—mainly Firmicutes—declines, while the proportion of Bacteroidetes and Proteobacteria increases. When microbiota communities are challenged by antibiotics, infections or other immune challenges, pathogenic bacteria can thrive and reshape the previous microbial community, potentially leading to dysbiosis [79,80]. The disturbed ecological dynamics occurring during ageing may allow for the evolution of novel—potentially threatening—bacterial strains [81].

Inflammaging and altered microbiota communities are mechanistically connected and the accumulation of pathogenic bacteria can lead to chronic intestinal inflammation, as in ulcerative colitis [82]. Loss of the transcription factor FoxO, which plays an important role in innate immunity, leads to diminished antimicrobial peptide expression and impaired selection of bacteria in *Hydra* [83]. In *Drosophila*, FoxO also regulates innate immunity and chronic FoxO activation (e.g. during ageing) is associated with deregulated immune function, disorganization of the fly gut epithelium (metaplasia) and bacterial dysbiosis [84]. Conversely, preventing age-related inflammation via immune-modulation maintains a healthy-like microbiota and prolongs lifespan in flies [69]. Similarly, during vertebrate ageing, the intestinal epithelial barrier becomes leaky and permissive for microbial invasion of the lamina propria through the basement membrane. Invasion of the lamina propria by luminal bacteria can lead to inflammation bursts and systemic inflammatory responses [82]. Further supporting the functional relevance of microbial communities during ageing, studies in naturally short-lived turquoise killifish have shown that the age-related decline in microbiota diversity was prevented by transfer of young-associated intestinal communities into middle-aged fish, leading to lifespan extension and delayed behavioural decline [71]. Note, transferring microbiota from old individuals to young-adult subjects did not lead to shortened lifespan in killifish, suggesting higher resilience in healthy individuals [71].

## 5. Immune system–microbiota interactions during vertebrate ageing

The immune system helps maintain systemic homeostasis and healthy tissues by eliminating damaged, infected and senescent cells [85] and by holding the balance between commensal and pathogenic microbes [86]. However, immune function is subject to severe alterations during ageing, including improper immune surveillance of damaged and infected cells, chronic inflammatory responses and autoimmunity, leading to extensive tissue damage [87]. As a consequence, during ageing the host is predisposed to infections and to a broad spectrum of diseases. Both innate and adaptive immune functions undergo changes during ageing. For instance, work in mice has shown that intracellular anti-viral mechanisms (e.g. interferon responses) become activated during ageing in response to de-repression of LINE1 (L1) retrotransposons. Cells sense cytoplasmic L1 DNA as a potential viral threat, activate inflammatory responses and induce cellular senescence [88], which plays a major role in ageing-related pathology [89].

Many aspects of adaptive immunity are also affected during ageing. Loss of B cell diversity [90] and lower quality of produced antibodies, including excess of non-specific antibodies [91], result in age-related immune dysfunction, which may for instance contribute to less efficient vaccination in the elderly in our species [92]. During human ageing, T cell mediated immunity is affected by reduced primary lymphopoiesis associated with thymus involution [93], and to a decreased diversity in the T cell receptor repertoire [94,95]. The functional decline of T cell responses during ageing has been linked with higher risk for cancer in the elderly [96].

The evolution of the adaptive immune system gave a formidable fitness advantage to early vertebrates, which could discriminate between self and non-self, effectively eliminating pathogens and parasites, becoming able to establish a diverse community of commensals. However, if the adaptive immune system provided vertebrates with the new opportunity to have access to the biochemical diversity of complex microbiota, it also came with the cost of a new range of homeostatic failure modalities, which we propose may define vertebrate-specific ageing dynamics.

Owing to the access to clean water, vaccinations and antibiotics, infectious diseases are no more the leading causes of death in industrialized countries. However, still today, the elderly are more susceptible to infections compared to young-adult individuals [97]. Immune decline associated with autoimmune diseases [98], pathogen-driven immunodeficiencies (e.g. owing to HIV infection) [99] and ageing, are often associated with intestinal dysbiosis [100], which, together with age-dependent loss of intestinal barrier function, can lead to bacteraemia and sepsis, i.e. two of the major causes of death in elderly populations [97]. During ageing, the fine discrimination between self and non-self can become defective, leading to increased autoimmunity, potential tolerance of pathogens [101], and de-repression of pro-inflammatory responses against commensals. Impaired immune surveillance during ageing may lead to proliferation of opportunistic pathogens and to the expansion of pathobionts, i.e. commensal microbes under normal circumstances which can become pathogens [81]. Pathobionts, in turn, can also causally induce autoimmunity [102].

Here, we propose that vertebrate-specific ageing dynamics depend in part on the complex mechanistic interplay between microbiota and adaptive immunity, which has a long evolutionary history. Indeed, the adaptive immune system actively shapes commensal microbiota, which in turn contribute to immune system development and maintenance. Emerging recent scientific evidence indicates how the microbiota plays a causal role during ageing. Transplanting microbiota from young-adults to middle-aged individuals extends lifespan in a short-lived vertebrate model system, showing that young-associated gut microbes have cumulatively a pro-biotic action [71]. Analogously, transplanting microbiota from young mice to middle-aged mice boosted immune function, reactivating defective germinal centres in the gut [103]; and transplanting microbiota from healthy donors to progeroid (i.e. prematurely ageing) mice led to lifespan and healthspan extension [104].

Several open questions remain to be addressed. If microbiota causally affect the ageing process, do individual-specific ageing dynamics—e.g. higher or lower risk for ageing-related diseases—depend on individual microbiota composition? Can we adopt the microbiota as a diagnostic tool to predict individual disease risk? If each species has its own, species-specific microbiota—which result as a function of their habitat, lifestyle, evolutionary history and specific immune system function—does each species differ accordingly in its ageing-related pathologies? If commensal microbes can suppress immune responses and favour immune tolerance (e.g. via SCFA), can pathogens hijack commensal's molecular signalling to selfishly favour pathogenicity? How can we maintain host–microbiota balance over extended time and during immune ageing?

Together, the evolution of multicellular hosts and their microbial partners has led to the emergence of astonishing biological innovations, including a sophisticated adaptive immune system, which led to the opportunity to access a novel metabolic space—provided to the host by its commensal microbes—but also resulted in new modalities of homeostatic failure, which possibly characterize vertebrate-specific ageing dynamics.

## Data accessibility

This article does not contain any additional data.
